# A Pilot Randomized Controlled Trial (RCT) Evaluating the Efficacy of an Exosome-Containing Plant Extract Formulation for Treating Male Alopecia

**DOI:** 10.3390/life15030500

**Published:** 2025-03-20

**Authors:** Farahnaz Amini, Jing Ju Teh, Chung Keat Tan, Eugenie Sin Sing Tan, Edmond Siah Chye Ng

**Affiliations:** 1 Faculty of Medicine (Neurology), University of British Columbia, Vancouver, BC V6T 1Z4, Canada; farahnaz.amini@ubc.ca; 2 School of Healthy Ageing, Aesthetics & Regenerative Medicine, Faculty of Medicine and Health Sciences, UCSI University, Kuala Lumpur 56000, Malaysia; jingju.teh@gmail.com (J.J.T.); cktan@ucsiuniversity.edu.my (C.K.T.); eugenietan@ucsiuniversity.edu.my (E.S.S.T.)

**Keywords:** alopecia, *Ecklonia cava* extract, exosome, growth factors, Norwood grade androgenetic, *Thuja orientalis* leaf extract

## Abstract

**Background/Objectives**: Hair loss affects self-esteem, confidence, and psychological well-being. Exosomes, as molecular carriers of growth factors and active compounds, offer a promising treatment. This study evaluates the efficacy of an exosome formulation containing extracts from two known hair-regenerating plants, *Ecklonia cava* and *Thuja orientalis* (ECPE), for male pattern alopecia. **Methods**: A randomized controlled trial included 20 male participants with Norwood grade 2–3 androgenetic alopecia who were randomly assigned into two groups, placebo (0.9% sodium chloride) and ECPE, administered bi-weekly across four sessions. Evaluations included hair density measurements, adverse effect tracking, and self-assessments. **Results**: Most participants (55%) were aged 18 to 35, with 75% reporting hair loss for over a year and 80% noting scalp thinning. The hair counts showed no significant change in the placebo group from baseline to week 16 (Wilcoxon signed-rank test: V = 13.5, *p* = 0.163), while a significant increase was observed in the ECPE group (V = 0, *p* = 0.002). Between-group analysis revealed a significant difference in the hair count changes (Wilcoxon rank-sum test: W = 86.5, *p* = 0.006) with a large effect size (Cliff’s Delta: & = 0.73, 95% CI: 0.41–0.89), with the ECPE group showing higher median hair growth (9.5, IQR = 16.88) compared to the placebo group (1.5, IQR = 3.00). A Bayesian ANCOVA, adjusted for covariates (the father’s scalp hair condition, baseline hair count, and Norwood classification), showed no significant effect of these factors on the outcomes. **Conclusions**: These findings suggest that ECPE significantly improves hair regrowth compared to the placebo, with no notable adverse effects.

## 1. Introduction

Hair loss is a growing concern affecting individuals of all ages and genders, often leading to significant psychosocial distress. While multiple treatment modalities exist, their efficacy remains variable and often suboptimal. This highlights the increasing global demand for more effective, evidence-based hair restoration therapies. Among the various types of hair loss, androgenic alopecia (AGA) is the most common, characterized by a progressive shortening of the anagen (growth) phase and an extended telogen (resting) phase, leading to hair follicle miniaturization and eventual baldness. The pathophysiology of AGA involves complex interactions between the epithelial and mesenchymal cells, which regulate hair follicle development and growth. Dermal papilla cells (DPCs), specialized mesenchymal cells, play a crucial role in follicular epithelium function by controlling outer root sheath cell (ORSC) proliferation, differentiation, and migration. However, dysregulation of these cellular interactions contributes to hair follicle shrinkage and hair loss [1,2].

Conventional treatments for AGA, such as minoxidil and finasteride, primarily aim to delay hair loss and stimulate regrowth. However, these treatments have limited effectiveness and require long-term use to maintain results. Additionally, they are associated with undesirable side effects, including scalp irritation, dizziness, and reduced libido, leading to poor adherence among patients. The need for more effective and safer therapeutic strategies has driven interest in innovative treatment approaches such as exosome-based therapies. Exosomes, small extracellular vesicles ranging from 50 to 200 nanometers in size, have emerged as a promising cell-free regenerative therapy. They facilitate cell communication and play an essential role in cell proliferation, migration, and differentiation, making them highly relevant for tissue repair and regeneration. Compared to stem cell-based therapies, exosomes offer several advantages, including a reduced risk of tumor formation, lower immunogenicity, and greater stability [3]. Their ability to transport proteins, microRNA, RNA, DNA, and lipids makes them an ideal molecular delivery vehicle with potential applications in regenerative medicine. Exosomes have been widely explored in dermatology for their role in treating hyperpigmentation, skin aging, and wound healing [4,5,6,7]. Additionally, in vitro and animal studies have demonstrated their effectiveness in promoting hair follicle proliferation, prolonging the anagen phase, and preventing follicular miniaturization [8,9,10,11]. These findings suggest that exosomes provide a more targeted, stable, and efficient method of delivering therapeutic agents to hair follicles compared to conventional drug delivery systems.

Beyond exosomes, botanical extracts have also shown promise in hair regrowth. Ecklonia cava, a marine brown alga, and Thuja orientalis, a medicinal plant, have been studied for their ability to promote hair follicle regeneration through distinct mechanisms [12,13,14,15]. *E. cava* is rich in bioactive compounds such as phlorotannins and fucoidans, which exhibit potent antioxidant and anti-inflammatory properties. These compounds reduce oxidative stress in dermal papilla cells, counteract androgen-induced TGF-β1 secretion, and enhance hair follicle proliferation [14]. Additionally, the topical application of 0.5% *E. cava* extract has been shown to accelerate hair growth in animal models by inhibiting 5α-reductase, the enzyme responsible for converting testosterone into dihydrotestosterone (DHT), a key factor in AGA progression. Similarly, *T. orientalis* has been found to activate the β-catenin and Sonic hedgehog (Shh) signaling pathways, which are critical for follicular cell proliferation and differentiation. The topical application of *T. orientalis* extract has been shown to induce the anagen phase in telogenic C57BL/6N mice, leading to increased hair follicle size and density [15].

Despite the promising effects of *E. cava* and Thuja orientalis, limited research has been conducted on their combined use with exosome-based formulations for hair growth in human subjects. Given the potential benefits of exosomes as a delivery system and the proven hair-regenerating properties of these plant extracts, this study aims to evaluate the efficacy of an exosome-containing formulation enriched with *E. cava* and *T. orientalis* compared to a placebo in treating male pattern alopecia, specifically in individuals classified as Norwood grades 2–3. By integrating exosome technology with natural bioactive compounds, this research seeks to contribute to the development of innovative, evidence-based therapeutic strategies for hair loss treatment.

## 2. Materials and Methods

### 2.1. Study Design and Study Subjects

A randomized, double-blind, placebo-controlled study was conducted to evaluate the efficacy of an intervention in middle-aged male participants diagnosed with Norwood grade 2–3 androgenic alopecia. The participants received detailed information through a participant information sheet and a thorough explanation from the investigators. Written informed consent was obtained from each participant. This study adhered to the ethical principles outlined in the Declaration of Helsinki and the Malaysian Guidelines for Good Clinical Practice. Ethical approval was granted by the UCSI Institutional Ethics Committee (IEC) under the approval code UCSI-IEC-2022-FMHS-062.

**Figure 1:**
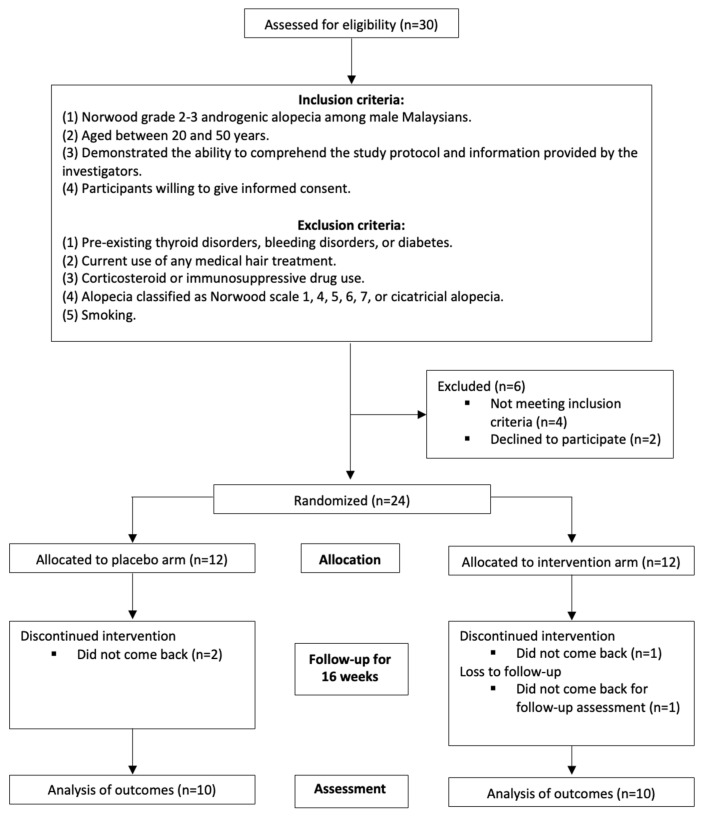
CONSORT protocol for study described with flow diagram.

Following sample size recommendations, a minimum of 10 participants per group were required for this pilot randomized trial [16]. To minimize the confounding variables, strict exclusion criteria were applied. Participants were excluded if they had pre-existing thyroid disorders, bleeding disorders, or diabetes, were currently using any medical hair treatments, were on corticosteroids or immunosuppressive drugs, or had alopecia classified as Norwood scale 1, 4, 5, 6, 7, or cicatricial alopecia. Additionally, individuals who smoked were deemed ineligible (Figure 1).

Of the 30 individuals assessed for eligibility, six participants were excluded—four for not meeting the inclusion criteria and two who declined participation. The remaining 24 eligible participants were randomized into two groups: 12 assigned to the placebo arm and 12 to the intervention arm. During the 16-week follow-up period, two participants from the placebo group discontinued the intervention, while two from the intervention group were lost to follow-up (one discontinued the intervention, and one did not return for the follow-up assessment). Consequently, the outcome analysis was conducted on 10 participants in each group (Figure 1).

To ensure unbiased allocation, the Randomization Envelope Seal Method was meticulously implemented. A total of 24 participants were randomly assigned into two groups (Group A and Group B). The randomization process was designed to uphold fairness and minimize bias. Randomized sequences were generated in advance to determine the group assignments. Each group was assigned a unique identifier, which was enclosed in sealed envelopes to maintain the allocation concealment. Secure sealing techniques were employed to preserve the integrity of assignments, with detailed documentation maintained throughout the process. The envelopes were only opened under controlled conditions by authorized researchers.

The study employed a double-blind design, ensuring that neither the participants nor the clinicians involved in the treatment administration or outcome assessment were aware of the group allocations. This rigorous blinding process minimized performance and detection biases, thereby enhancing the validity and reliability of the findings. The randomized group allocation and blinding procedures were independently prepared and labelled as Group A and Group B by Cosmedician AP Sdn Bhd, Subang Jaya, Malaysia.

### 2.2. Intervention Plan

The participants were randomized into two groups: the control group (Group A), which received 0.9% sodium chloride as a placebo, and the intervention group (Group B), which received an exosome formulation containing 10 billion exosomes combined with *E. cava* and *T. orientalis* leaf extracts.

All formulations were prepared by DASAN C&Tech CO. LTD., Gimpo-si, Republic of Korea, and packaged in 5 mL vials. The vials were stored under controlled conditions at 4 to 8 °C to maintain product stability.

The intervention protocol consisted of four sessions, each spaced 15 days apart, and was administered by a certified aesthetic physician in clinical settings. Preoperative dots were marked with a pen to delineate areas of hair loss or thinning, including the frontal–temporal zones, mid-scalp, and crown areas. Aseptic preparation involved the application of 70% isopropyl alcohol to the targeted area. To minimize the procedural discomfort, local anesthesia was achieved with 2% lidocaine, administered via the bilateral supratrochlear and supraorbital nerve blocks using a 30-gauge needle and a tuberculin syringe, with a total volume of 2 mL.

Following anesthesia, the treatment solution was drawn into five 1 mL tuberculin syringes, each fitted to a 30-gauge short needle. Intradermal injections of 0.05 to 0.1 mL per site were administered at approximately 1 cm apart, delivering a total volume of 5 mL per session.

The participants were advised to maintain their daily routines or hair care practices to minimize confounding variables that could influence the treatment outcomes.

### 2.3. Outcome Measurements

The hair measurement tools utilized in this study included the Canon EOS 200D II digital camera (Canon, Taichung, Taiwan, China) for standardized profile photography, capturing lateral, facial, occipital, and cephalic views at baseline, 12 weeks, and 16 weeks post-treatment. Additionally, the Dino-Lite Trichoscope (Dino-Lite Europe, Almere, The Netherlands) was used to capture high-resolution images at a standardized anatomical reference point, defined as the intersection of a vertical line extending from the lateral canthus of one eye (either left or right) and a coronal line connecting both ears. Hair density was assessed by counting the number of hairs within a 0.5 cm^2^ circular area, with the evaluations conducted by a blinded medical assessor. Trichoscopic assessments were performed at baseline, 12, and 16 weeks post-treatment to monitor changes in hair density and scalp condition.

Secondary outcomes included the participants’ self-perceived satisfaction with the overall hair growth improvement, assessed using a validated questionnaire with five statements rated on a 5-point Likert scale. Also, the participants completed a supplementary questionnaire to evaluate their dietary habits, medical history, hair care routines, and family history of hair loss.

### 2.4. Statistical Analysis

The data were imported into R Studio Version 4.0.5 (R Foundation, Vienna, Austria) and cleaned for analysis. Descriptive statistics was initially employed to summarize the demographic and baseline characteristics of the participants across the two groups, with the continuous variables presented as medians and interquartile ranges (IQRs). Hair growth was quantified based on the change in hair count from baseline to week 16, with the mean change in hair number used as the primary outcome measure. The normality of the hair growth distribution within each treatment group was assessed by the Shapiro–Wilk test. As the data exhibited a non-normal distribution, a Wilcoxon rank-sum test (Mann–Whitney U test) was used to compare the hair growth between the placebo and the treatment group. The participants’ self-perceived satisfaction scores were analyzed using the independent T-test. The effect size was estimated using Rank-Biserial Correlation (r) with interpretation based on the conventional guidelines: small (r = 0.1–0.3), moderate (r = 0.3–0.5), and large (r > 0.5). To adjust for potential confounders, ANCOVA was conducted, controlling for variables including Norwood classification, father’s and mother’s scalp conditions, and duration at which hair loss was first noticed.

## 3. Results

### 3.1. Participant Characteristics and Baseline Data

This study initially recruited 24 participants; however, due to participant dropout and loss to follow-up, only 20 participants were included in the final analysis. The majority (56%) of the participants were aged 20 to 35 years, followed by 37% in the 36- to 50-year age group and 7% over 50 years old. Most participants (75%) noticed hair loss for over a year, with scalp thinning being the most commonly observed sign (80%). Additional data on hair loss onset, progression, contributing factors, and hair care practices are listed in Table 1. All participants reported washing or shampooing their hair daily. Except for one participant who reported an iron deficiency, none underwent blood or hormone tests to assess potential abnormalities associated with their hair loss. The family histories revealed that most participants’ fathers had some degree of hair thinning, as detailed in Table 2.

### 3.2. Hair Growth Outcomes

Throughout the study period, the effectiveness of the treatment was evaluated by calculating the mean number of hair strands through photographic documentation and trichogram analysis conducted at week 0, week 12, and week 16 in each group. The baseline hair counts did not significantly differ between the ECPE and placebo groups (Kruskal–Wallis test: χ^2^(2) = 4.745, *p* = 0.093). The Shapiro–Wilk normality test indicated that the hair growth data were not normally distributed (ECPE: W = 0.831, *p* = 0.035; placebo: W = 0.612, *p* < 0.001), justifying the use of non-parametric statistical tests for further analysis.

Within-group comparisons showed no statistically significant change in hair counts from baseline to week 16 in the placebo group (Wilcoxon signed-rank test: V = 13.5, *p* = 0.163). However, the treatment group demonstrated a statistically significant increase in hair counts over the same period (Wilcoxon signed-rank test: V = 0, *p* = 0.002).

**Figure 2:**
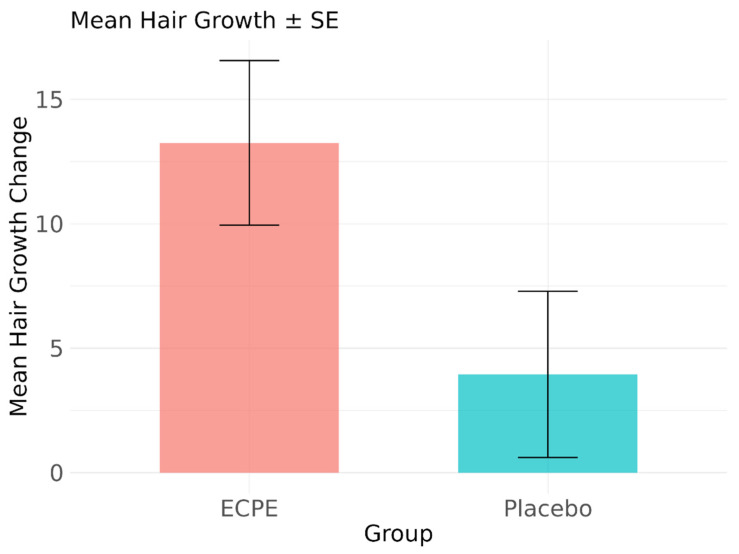
Comparison of mean hair growth changes between treatment groups. Bar chart showing mean change in hair growth from baseline to week 16 for ECPE and placebo groups. Error bars represent standard error of mean (SE).

Between-group analysis showed a significant improvement in the ECPE group compared to the placebo group (Wilcoxon rank-sum test: W = 86.5, *p* = 0.006) (Figure 2). The median hair growth was 9.5 (IQR = 16.88) in the ECPE group and 1.5 (IQR = 3.00) in the placebo group.

Similarly, the self-perceived satisfaction scores among the participants reflected comparable outcomes, with the ECPE group demonstrating significantly higher satisfaction (*p* < 0.05) compared to the placebo group (Table 3).

### 3.3. Trichoscopic and Photographic Assessments

**Figure 3:**
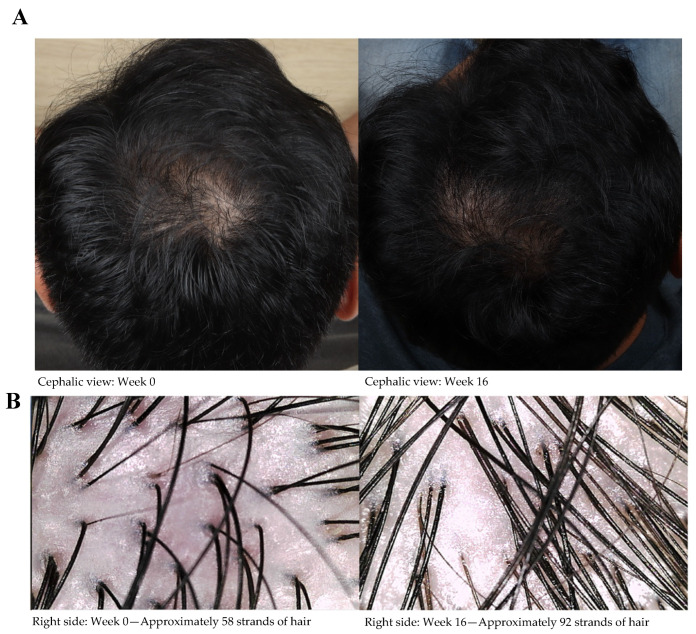
(**A**) Camera photos of the cephalic view of a participant treated with ECPE at weeks 0 and 16, and (**B**) trichoscopic images of the right side of the scalp of the same participant at weeks 0 and 16.

The photographic and trichoscopic evaluations further supported the observed hair growth improvements. Figure 3A shows standardized cephalic view photographs of a participant’s scalp at baseline and week 16, demonstrating visible improvements in hair density. The trichoscopic images in Figure 3B highlight the increased hair strand counts in the ECPE group after 16 weeks.

### 3.4. Statistical Analysis and Effect Size

An ANCOVA analysis was conducted to assess the influence of the covariates on hair growth. While the main effect of the treatment remained significant, none of the covariates, including baseline hair counts, Norwood classification, and parental scalp hair status, significantly influenced the primary outcome (*p* > 0.05 for all covariates). Cliff’s Delta indicated a large effect size (δ = 0.73, 95% CI: 0.41–0.89), suggesting that the participants in the ECPE group experienced notably higher hair growth compared to those in the placebo group.

### 3.5. Adverse Events and Safety Profile

No severe adverse effects were reported during the study. Mild scalp irritation was observed in two participants from the ECPE group, which resolved spontaneously without intervention.

## 4. Discussion

This study demonstrated that an exosome formulation containing *E. cava* extract and *T. orientalis* leaf extract (ECPE) significantly improved hair growth in male participants with Norwood grade 2–3 androgenetic alopecia over 16 weeks. The ECPE group exhibited a statistically significant increase in hair counts compared to baseline, while no significant change was observed in the placebo group. The between-group analysis confirmed a significant difference in hair growth favoring the ECPE group, with a large effect size, highlighting the potential of exosome-based formulations in promoting hair regeneration. Given alopecia’s psychological and physical impacts and the limitations of current treatments like minoxidil and finasteride, including side effects and long-term challenges, there is a growing demand for effective therapies [17]. Unlike these treatments, exosomes offer a cell-free option, potentially avoiding the immunogenicity and tumorigenicity concerns associated with stem cell therapies. The significant hair growth observed in this study suggests exosomes may recalibrate the hair growth cycle, shifting follicles from dormancy to active growth, paving the way for future research on their mechanisms in androgenetic alopecia. In concurrence with the beneficial effects of exosomes, the participants in the ECPE group also reported significantly higher self-perceived satisfaction scores compared to the placebo group (*p* < 0.05). This suggests that the intervention effectively enhanced participant satisfaction, potentially due to improved engagement, perceived benefits, or overall experience [18]. These results align with previous studies highlighting the importance of effective treatment in improving self-confidence and quality of life [19].

As cell-derived vesicles, exosomes have attracted much attention for their role in cell communication and their ability to deliver molecular signals that can influence hair follicle growth and regeneration. The targeted delivery of growth factors, natural compounds, and proteins via exosomes could mimic the natural growth signals that are lessened in balding scalps. Pre-clinical studies have demonstrated the effectiveness of mesenchymal stem cell-derived exosomes in stimulating hair growth and the migration and proliferation of hair-specific fibroblasts, and in modulating the cell signaling pathways necessary for hair follicle morphogenesis and growth [8]. Despite the promising evidence, there is still a gap in the number of human clinical trials confirming the efficacy and safety of exosome therapy for hair loss treatment.

Adipose-derived stem cell-conditioned media were combined with minoxidil to treat male androgenetic alopecia in a randomized, double-blind trial including 37 men aged 25–59, and significant hair regrowth was found on both the treated and placebo sides of the head, with no significant differences between groups [20]. Similarly, exosomes derived from dermal papilla (DP) cells promoted hair growth by accelerating the telogen-to-anagen transition and delaying the anagen-to-catagen phase [11]. Expressing the CD9, CD63, and TSG101 markers increased the outer root sheath cell proliferation, migration, and cell cycle progression through β-catenin and Sonic hedgehog signaling. Exosomes from 3D-cultured DP cells further stimulated DP and ORS cell proliferation, increased growth factor expression (IGF-1, KGF, HGF), and promoted hair shaft elongation in human hair follicles. Also, local injections in mice induced and prolonged the anagen phase [21].

Exosomes derived from human hair outer root sheath cells (HHORSCs) and platelet lysis (PL) have also shown potential in enhancing the hair inductivity of human dermal papilla cells (hDPCs) by promoting proliferation and migration and upregulating key genes like ALP, versican, and alpha-SMA [22]. Similarly, adipose-derived stem cell (ADSC) exosomes, when applied to nude mice during grafting, significantly increased hair regeneration, as evidenced by a higher number of regenerated follicles and elevated expression of growth factors like PDGF and VEGF, while reducing TGF-beta1 levels [23]. Additional evidence supports the efficacy of exosome-based therapies for hair regeneration. A case study reported successful hair regrowth and repigmentation in a 38-year-old male with androgenetic alopecia and poliosis circumscripta using exosomes and fractional picosecond laser treatment [24]. Similarly, a large-scale study involving over 1000 alopecia patients demonstrated significant hair growth with adipose-derived stem cell-conditioned medium (ADSC-CM) injections containing VEGF, HGF, bFGF, KGF, and PDGF, administered monthly for 6–8 sessions with no significant adverse effects [25]. Another study with 25 alopecia patients showed that hypoxia-cultured ADSCs enriched with essential growth factors and nutrients, applied via mesotherapy techniques, resulted in sustained hair regrowth for 1.5 to 2.5 years and increased patient satisfaction over time [26].

## 5. Conclusions

This study confirmed the potential of exosome-containing hair formulations as an alternative and effective treatment for male androgenetic alopecia. The findings add value to the evidence supporting exosome-based formulations in promoting hair regeneration and growth, particularly when combined with bioactive compounds. However, given the small sample size and short follow-up period, the generalizability of these results should be approached with caution. Further research with larger sample sizes and extended follow-up periods is required to confirm these results, assess the long-term efficacy, and monitor potential side effects. Further investigation is needed to elucidate how exosomes stimulate hair follicles and optimize key therapy parameters, including the dosage, formulation, and treatment frequency.

## Figures and Tables

**Table 1:** Onset, progression, and potential contributing factors of hair loss.

No.	Question	Number (%)
1	When did you first notice that you were losing your hair?	
	Less than a year	5 (25%)
	More than a year	15 (75%)
2	What did you notice at that time?	
	Hair coming out	3 (15%)
	Shedding	1 (5%)
	Hair looked thinner on scalp	16 (80%)
3	Have you recently noticed that your hair loss was worsening?	
	Yes	12 (60%)
	No	8 (40%)
4	Have you had a serious illness at any time before or during the hair loss?	
	Yes	0
	No	20 (100%)
5	Have you been hospitalized at any time before or during the hair loss?	
	Yes	1 (5%)
	No	19 (95%)
6	Have you started any special diets at any time before or during the hair loss?	
	Yes	0
	No	20 (100%)
7	Have you been under a severe amount of stress at any time before or during the hair loss?	
	Yes	6 (30%)
	No	14 (70%)
8	Heat-processed hair treatment	
	Never	20 (100%)
	Once every 1–2 months	0
9	Chemical hair processing and straightening	
	Never	20 (100%)
10	Color treatment	
	Never	18 (90%)
	Once a week	0
	Once every 2–3 weeks	1 (5%)
	A few times a year	1 (5%)

**Table 2:** Family history of hair loss.

Condition	Father	Mother	Brother(s) *	Sister(s) **
Has a lot of hair	1	6	2	3
Has some thinning	17	9	6	3
Has a small bald area	1	5	8	0
Has a large bald area	1	0	0	0
Has many bald spots	0	0	0	0

* 4 participants had no brothers. ** 14 participants had no sisters.

**Table 3:** Participants’ self-perceived satisfaction scores.

Variable	Placebo	ECPE	*p*-Value
Self-perceived satisfaction scores	2.90 ± 0.994	4.20 ± 0.632	0.013 *

Statistically significant *p*-values are marked in asterisks (*).

## Data Availability

The datasets generated during and/or analyzed during the current study are available from the corresponding authors on reasonable request. All of the individual participant data collected during the trial, after deidentification, will be shared upon reasonable request. Additional documents including study protocols, statistical analysis plans, informed consent forms, and clinical study reports will also be made available. The data will be available immediately following publication, with no end date. The data will be shared with anyone who wishes to access them on reasonable request. The data can be used for any type of analysis.

## Abbreviations

The following abbreviations are used in this manuscript:

ADSC	Adipose-derived stem cell
AGA	Androgenic alopecia
ANCOVA	Analysis of covariance
bFGF	Basic fibroblast growth fact
DNA	Deoxyribonucleic acid
DP	Dermal papilla
DPCs	Dermal papilla cells
ECPE	*Ecklonia cava* and *Thuja orientalis*
hDPCs	Human dermal papilla cells
HGF	Hepatocyte growth factor
HHORSCs	Human hair outer root sheath cells
IGF-1	Insulin-like growth factor 1
IQR	Interquartile range
KGF	Keratinocyte growth factor
microRNA	Micro ribonucleic acid
ORS	Outer root sheath
ORSCs	Outer root sheath cells
PDGF	Platelet-derived growth factor
PL	Platelet lysis
RNA	Ribonucleic acid
TGF-β1	Transforming growth factor beta
VEGF	Vascular endothelial growth factor
